# Total Phenolic, Flavonoid Content, and Antioxidant Activity of Dried Marigold (*Tagetes erecta* L.) Petals Produced in a Mixed-Mode Solar Dryer

**DOI:** 10.1007/s11130-025-01366-z

**Published:** 2025-05-17

**Authors:** Alfredo Domínguez-Niño, Paulina Guillén-Velázquez, Iris Santos-González, Octavio García-Valladares, José Manuel Vázquez-Morales

**Affiliations:** 1 Departamento de Sistemas Energéticos, Instituto de Energías Renovables-UNAM, Temixco, Morelos México; 2 Secretaría de Ciencia, Humanidades, Tecnología e Innovación, Dirección Adjunta de Desarrollo Científico Mexico City, Mexico City, México; 3 Secretaría de Ciencia, Humanidades, Tecnología e Innovación, Estancias Posdoctorales Por México, Mexico City, México; 4 Secretaría de Ciencia, Humanidades, Tecnología e Innovación, Investigadoras E Investigadores Por México, Mexico City, Mexico

**Keywords:** *Tagetes erecta*, Solar energy, Bioactive compounds, Solar drying, Color

## Abstract

Marigold (*Tagetes erecta L.*), commonly known as Cempasúchil, is a significant symbol of Mexico's Day of the Dead celebrations. However, once the festivities conclude, the flowers and petals are frequently discarded as waste, contributing to environmental pollution. Cempasúchil was dehydrated using an active mixed-type solar dryer to address this issue. This method establishes an environmentally friendly, easy-to-use, cost-effective, and practical preservation technique for rural communities. The maximum drying temperature was 69.65 °C, and the maximum solar irradiance was 1080.60 W/m^2^. The initial moisture content began at 87.35% and finished at 4.93%. The initial colorimetric parameters were: lightness 65.44, a 32.43, b 78.38, Chroma 84.83, and Hue angle 67.53°. The antioxidant activity of the marigold flower was 93.51%, ascorbic acid 27.86 mg/100 g, total phenols content 28.06 mg GAE/g dry mass (gallic acid equivalents), total flavonoids content 6.34 QE/g (quercetin equivalent) and carotenoid content 2.07 mg/g. After drying it was observed an increment in vitamin C (from 27.86 to 75.675 mg/100 g), carotenoids content (from 2.073 to 18.947 mg/100 g.), and antioxidant activity (from 93.513 to 98.178%). No significative changes in total phenols content with a value of 28.488 QE/g for dried marigold samples and flavonoid content from 6.348 QE/g and 6.622 QE/g were observed. Dried flowers and petals can be used in practical applications, such as in agriculture. They serve as a natural insecticide, providing an alternative for pest management, and can also act as a fertilizer, enhancing the soil's physical, chemical, and biological conditions.

**Supplementary Information:**

The online version contains supplementary material available at 10.1007/s11130-025-01366-z.

## Introduction

Marigold, *Tagetes erecta* L. [1], also known as cempasúchil, from the Nahuatl cempohualxochitl, which means twenty flowers, is an annual plant [2], up to 120 cm tall, vastly aromatic when squeezed, yellow to orange color. It belongs to the *Tagetes* genus, which has about 55 species throughout the American continent and 8 in the valley of Mexico [3]. Cempasúchil is endemic to Mexico [2] and has been registered in the states of Zacatecas, Aguascalientes, Yucatán, Campeche, Tlaxcala, Ciudad de México, Tamaulipas, Chiapas, Tabasco, Coahuila, Sonora, Colima, Sinaloa, Durango, San Luis Potosí, Estado de México, Quintana Roo, Guerrero, Querétaro, Hidalgo, Puebla, Jalisco, Oaxaca, Michoacán, Nayarit, Morelos, and Veracruz [4]. *Tagetes erecta* L., is one of Mexico's most representative icons of the day of the Dead festivities. They are easy to grow and do not require much water. According to Mexican tradition, these flowers decorate altars, and their petals are used to create paths that guide souls from the home's entrance to the altar. In 2023, more than 5 million plants were cultivated, with a sales percentage of 95% [5]. However, once the festivities are over, the flowers and petals are often discarded as waste, contributing to environmental pollution. Some media outlets estimate that tons of waste are collected after cleaning activities at municipal cemeteries. However, Mexico has no precise data on the amount of waste generated nationwide. Currently, recycling and reuse initiatives have been implemented, establishing collection centers. However, these efforts are still limited. Dried flowers and petals can be used in practical applications such as agriculture; they can be used as a natural insecticide since they represent an alternative for pest management and as a fertilizer, improving the physical, chemical, and biological conditions of the soil, in addition to providing nutrients to crops [6–9]. Likewise, its use facilitates the extraction of pigments [10], which are used as dyes in the food and textile industries. *Tagetes erecta* L. has little-known properties that can be used in various ways. In Mexico, it also has medicinal uses, and like most medicinal plants, all parts of it (root, stem, and flowers) are used. *Tagetes erecta* L. treats colds, bronchitis, stomach aches, intestinal diseases, diarrhea, liver problems, indigestion, colic, and menstrual irregularities [11]. In other parts of the world, *Tagetes erecta* L. has other medicinal uses, such as treatment for burns, wounds, ulcers, kidney problems, and muscular pain [12]. It is also used as a carminative, vermifuge, diuretic, and root infusion as a laxative. The pigments extracted from the plant are used in food, like eggs with yellow yolk, pastas and yellow skin and fat of birds. Moreover, the plant contains flavonoids, phenolic metabolites, and carotenoids like lutein, which can explain its medicinal uses [13]. In particular, lutein has been reported to protect against atherosclerosis, cardiovascular diseases, and cancer. In addition to its medicinal uses, *Tagetes erecta* L. is also used as food; its flowers are included in salads and are one of the world's most popular edible flowers. [14]. However, edible flowers are perishable and have a very short shelf life; hence, extending their shelf life and conserving the bioactive compounds is important. Drying is the simplest and most basic method for preserving edible flowers, as it removes water and moisture, thus avoiding degradation caused by enzymes and microorganisms. There are various techniques for drying them, but solar drying stands out as an efficient alternative, as it is easy to implement, requires little or no electricity, produces high-quality products, and has lower operating costs and a reduced environmental impact. A solar dryer reduces product moisture through natural ventilation, forced convection, and solar radiation. These dryers are divided into two types: active and passive. In active systems, fans are used inside or outside the cabinet to improve air circulation in the drying chamber. In passive systems, on the other hand, the air is heated and circulated naturally thanks to wind pressure and/or buoyancy force. Active and passive dryers are classified into direct, indirect, and mixed-mode subcategories. In direct dryers, the transparent drying chamber allows solar radiation to hit the products, which can modify their color. The system has a solar collector and an opaque drying chamber in indirect dryers. The hot air generated in the collector is moved into the chamber, preventing the products from receiving direct solar radiation, which helps preserve their color and reduces the formation of cracks. Finally, mixed-mode dryers combine characteristics of indirect dryers, as they include a solar air heater (solar collector), but their drying chamber has transparent walls, allowing a portion of the solar radiation to reach the products [15]. This research used a mixed-mode solar dryer to evaluate the effects on the physicochemical characteristics of dried marigold flowers. In the literature, some researchers have reported the solar drying of edible flowers by using solar technology: jasmine (natural convective solar drying) [16], pumpkin flower (direct cabinet solar dryer) [17], zompantle (mixed mode solar drying) [18], rose (using the solar wire basket dryer) [19], rose (hybrid solar dryer) [20], however, the study of drying process of marigold by using new cheap and easy to use solar technology has not been done.

## Materials and Methods

A detailed description is provided in the supplementary information file.

## Results and Discussion

### Drying Kinetics and Characterization of Marigold Flower

**Fig. 1:**
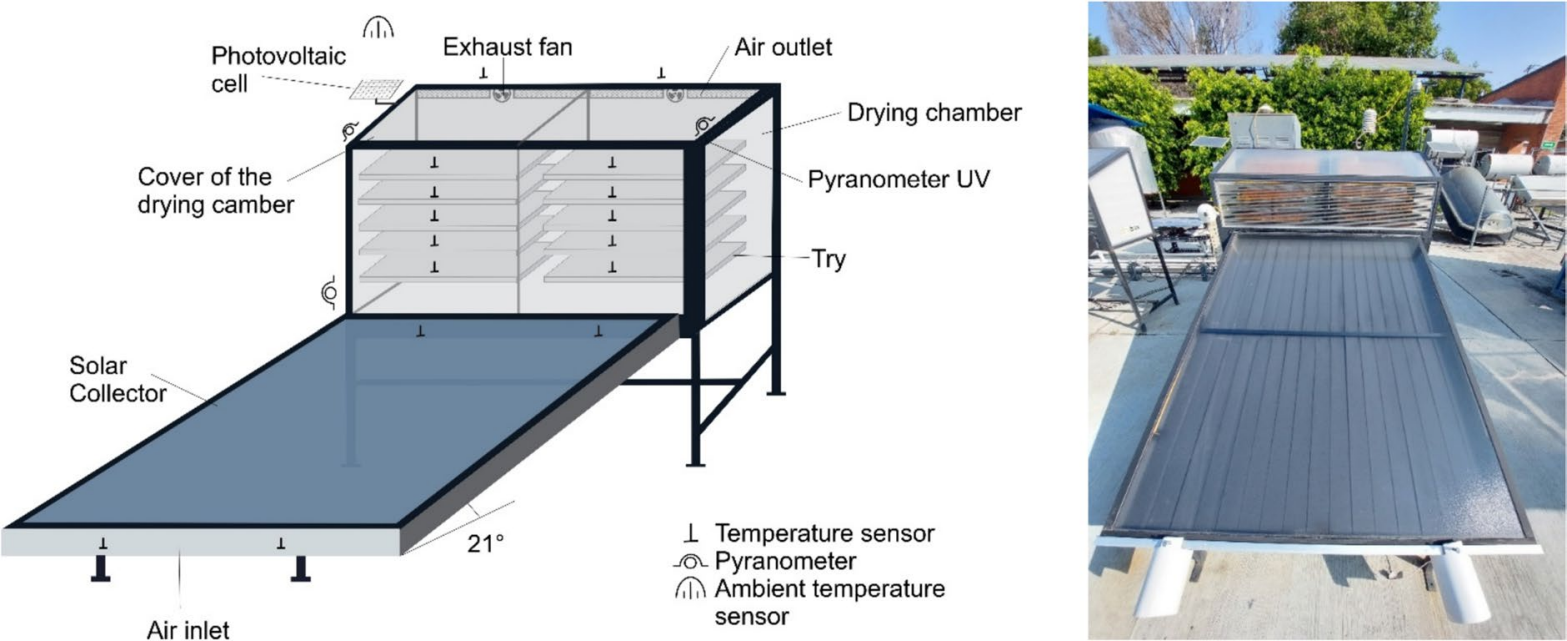
Mixed-type solar dryer used in the drying process of Marigold (*Tagetes erecta* L.)

The drying kinetics of marigold flowers were carried out on 21 October 2024 and 24 October 2024 (Supplementary Fig.1 A and B) using a mixed-mode solar dryer (Fig. 1). The test began from 09:00 to 14:00 h.

**Fig. 2:**
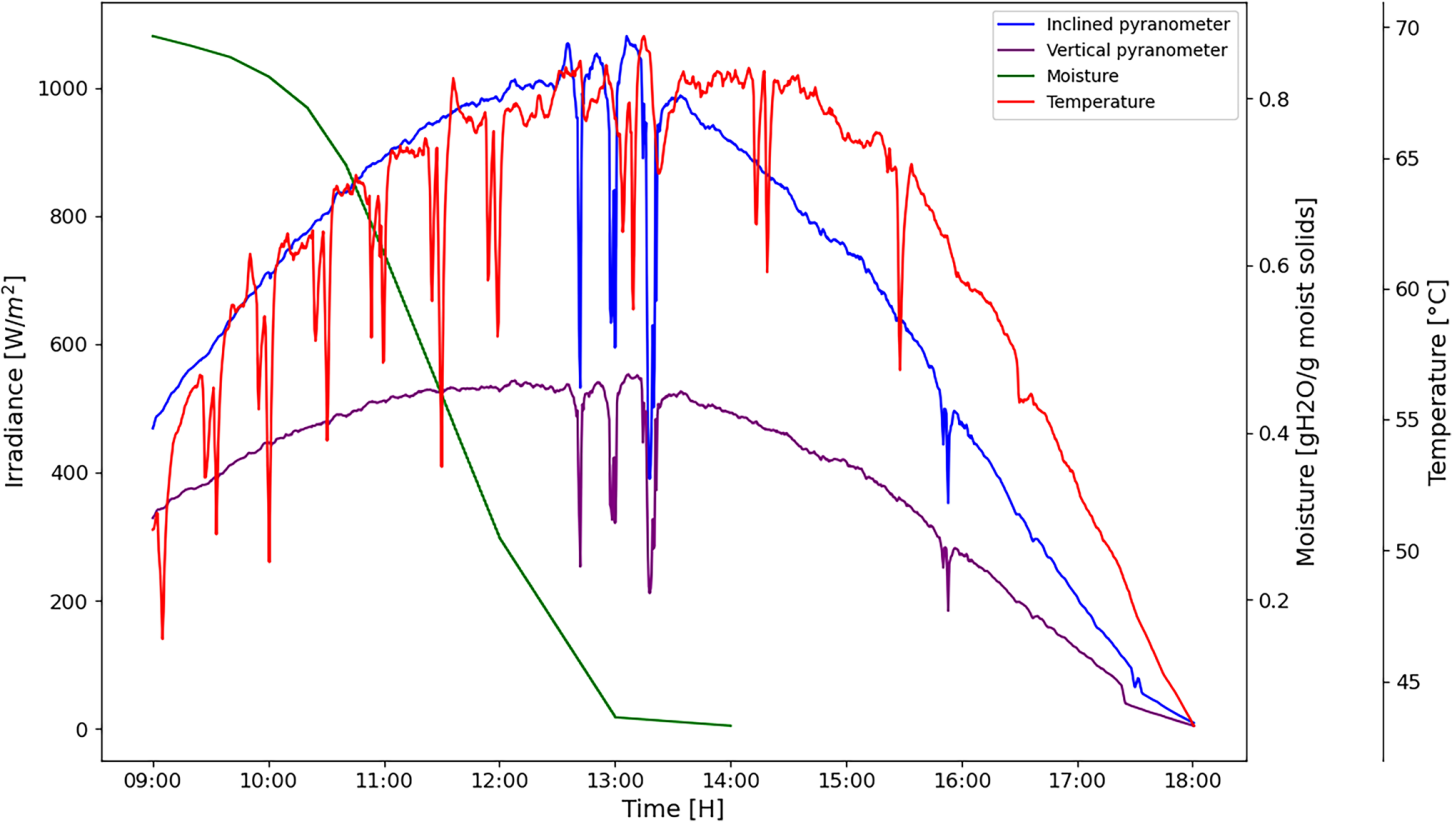
Drying kinetics of marigold (*Tagetes erecta* L.) carried out in a mixed mode solar dryer on the day 21 October 2024

The initial moisture content began at 87.35% and finished at 5.687%. Sowbhagya [21] reported 93% of moisture content in marigold flowers. The maximum drying temperature was 69.65 °C, and the maximum solar irradiance was 1080.60 W/m^2^ (Fig. 2).

The initial water activity was reduced from 0.96 to 0.36. The initial colorimetric parameters were as follows: lightness (*L)* 65.44, *a* 32.43, *b* 78.38, Chroma (*c*) 84.83, and Hue angle (*H*) 67.53 (Table 1).

**Table 1:** Physicochemical analysis of fresh and dried marigold flower (*Tagetes erecta L.*)

Analysis	Fresh Marigold flower	Dried Marigold flower
Moisture content (w.b)	87.35^±0.142^	5.687^±0.289^
Water activity	0.962^±0.010^	0.368^±0.027^
Antioxidant activity (%)	93.513^±0.309^	*98.178^±0.001^
Ascorbic acid (mg/100 g)	27.86^±2.539^	*75.675^±1.513^
Total phenols content (mg GAE/g dry mass)	28.06^±1.426^	28.488^±0.059^
Total flavonoids content (QE/g)	6.348^±0.127^	6.622^±0.333^
Carotenoid content (mg/g)	2.073^±0.041^	*18.947^±0.379^
Color parameters	L 65.44^±0.084^	ΔL −15.51^±2.679^
	a 32.43^±2.333^	Δa −1.56^±0.014^
	b 78.38^±3.026^	Δb −17.5^±0.685^
	C 84.83^±3.691^	ΔC −16.57^±0.601^
	H 67.53^±0.685^	ΔH −5.85^±0.332^
		ΔE 23.44^±2.05^

*L* (Lightness), *a* (Green–Red), *b* (Yellow-Blue), ΔL (Lightness difference), Δa (Redness-greenness difference), Δb (Yellowness-blueness difference), ΔC (Chroma difference), ΔH (Hue angle difference), ΔE (Color difference), Mean values^±^ Standard deviation, *Mean values are significantly different from control (fresh marigold flower)

As seen from the results, the redness is represented by *a* on the positive side, and the yellowness is represented by *b* on the positive side; this means that the color tends to be yellow-reddish. Dujmovic [22] reported the chromaticity parameters of fresh edible flowers as common marigold (lightness 70.90, *a* 26.48, *b* 73.14, Chroma 80.12 and Hue angle 72.89°) and African marigold (lightness 38.40, *a* 25.54, *b* 37.79, Chroma 53.90 and Hue angle 67.17°); Siriamornpun [23] reported the color of marigold flower; according with their results, the lightness was 83.2, *a* −3.4, and *b* 68.8, *as* seen from the results, the difference between the parameters values depending on the flower species. In this study, the antioxidant activity of marigold flower was 93.51%, ascorbic acid 27.86 mg/100 g, total phenols content 28.06 mg GAE/g dry mass, total flavonoids content 6.34 QE/g and carotenoid content 2.07 mg/g. Dujmovic [22] reported the antioxidant capacity of common marigold (2489.04 µmol TE/L), 25.46 mg/100 g of ascorbic acid, 379.38 GAE/100 g of total phenols content, 165.49 CTH/100 g of total flavonoid content and 0.42 mg/g of total carotenoid content; on the other hand, the African marigold showed 2500.22 µmol TE/L of antioxidant activity, 36.69 mg/100 g of ascorbic acid, 898.19 mg GAE/100 g of total phenols content, 391.09 mg CTH/100 g of total flavonoid content and 36.69 mg/100 g of ascorbic acid. The results show that the difference between the parameter values depends on the flower species.

### Vitamin C

Vitamin C values of marigold flowers described an increase from 27.86 to 75.675 mg/100 g after the solar drying process (Table 1). A similar behavior was reported by García [17] in the pumpkin flower solar drying with an initial value of 3.5 mg/100 g to a range between 5.87 and 8.806 mg/100 g. Another product that reported a considerable increment in this parameter was carambola fruit, from 44.03 mg/100 g to 124.37–159.5 mg/100 g [24]. The authors explain that vitamin C is stable at low water activity; in this sense, low water activity levels after drying (0.368) allow the preservation and enhancement of vitamin C values. On the other hand, Shi [25], reported a degradation in vitamin C at higher microwave power (510–850 W) and higher oven temperature (55, 65, and 75 °C) in medicinal chrysanthemum flowers; similar behavior was reported by Gargi [26] in the drying process of pumpkin flower (*Cucurbita maxima*), according to their results, the initial ascorbic acid in fresh pumpkin flower (27.6 mg/100 g) decreased to 18 mg/100 g in tray drying, 24 mg/100 g in shade drying, 17 mg/100 g in microwave drying, and 13.6 mg/100 g in sun drying process. Mashitoa [27] reported an initial ascorbic acid content of 62.37 mg/100 g in pumpkin leaves (*Cucurbita moschata*); however, during different drying treatments, the ascorbic acid content decreased to 46.24 mg/100 g in freeze-drying, 34.41 mg/100 g in microwave-drying, 26.88 mg/100 g in oven-drying, 39.78 mg/100 g in solar drying, and 37.63 mg/100 g in sun-drying.

### Carotenoid Content

Carotenoids present in cempasuchil flowers contribute to various important biological functions. Authors such as Vanegas [28] reported inhibition of colon cancer cell growth; carotenoids have been associated with preventing macular degeneration and cataracts. In this study, carotenoids increased significantly with the decrease in moisture content from 2.073 to 18.947 mg/g (Table 1). A similar behavior was reported by Sarkar [29], where African *Tagetes erecta* L. was dehydrated by several methods, including solar drying. Carotenoids found in cempasuchil flowers are a source of natural pigments that can be employed in the food industry and, due to their benefits to health consumers, as a dietary supplement. Mashitoa [27] reported a decrease in carotenoid content by using different drying treatments from 2.23 mg/100 g to 1.01 mg/100 g (freeze-drying), 0.11 mg/100 g (microwave-drying), 0.08 mg/100 g (oven-drying), 0.47 mg/100 g (solar-drying), 0.15 mg/100 g (sun-drying). On the other hand, Managa [30] reported an increment in carotenoid content during sun drying, solar drying, and freeze drying from 0.93 mg/100 g to 1.81 mg/100 g for Chinese cabbage and African nightshade, respectively. The changes in carotenoid content have been reported by Siriamornpun [23], Gargi [26], and Akshaya [31].

### Antioxidant Activity

Antioxidants in *Tagetes erecta* L. constitute an important component because they add value as a functional food and/or nutraceutical ingredient [14]. It has been reported that antioxidants can prevent and treat diseases by scavenging free radicals and regulating the activity of different types of oxidases in the body [32]. Results demonstrated an increase in antioxidant activity from fresh flowers (93.513%) to dried ones, with a value of 98.178% (Table 1). Siriamornpun [23] also reported an increment in the marigold flower's antioxidant activity after drying. In this investigation, they explain it due to increased lycopene, b-carotene, and lutein, a major phytochemical in marigold, and other bound phytochemicals released from the matrix with thermal processing. In this research, the increment in the percentage of inhibition of DPPH radicals is attributed to carotenoids and ascorbic acid release, resulting in a major value compared to fresh flowers. Managa [30] reported a decrease in antioxidant activity during sun and microwave drying; however, an increase was reported during the freeze-drying process of Chinese cabbage and Nightshade leaves. Mashitoa [27] reported the effect of different drying treatments on antioxidant activity of pumpkin leaves; according to their results, the antioxidant activity expressed in µmol TEAC 100 g^−1^ (Trolox Equivalent Antioxidant Capacity) increased from 23.35 to 25.01 µmol TEAC 100 g^−1^, 24.31 and 23.52 µmol TEAC 100 g^−1^ by using freeze drying, solar drying, and sun drying method, respectively; however, the antioxidant activity decreased from 23.35 µmol TEAC 100 g^−1^ to 22.04 and 10.18 µmol TEAC 100 g.^−1^ by using microwaved and oven drying, respectively. In most cases, drying reduces antioxidant content due to oxidation processes or thermal degradation. However, in some cases, increases have been observed, and some authors explain that it can be related to the Maillard reaction products, which can be formed because of heat treatment and also to the fact that partially oxidized polyphenols have more significant antioxidant activity than non-oxidized polyphenols [33]

### Total Phenols Content

Total Phenols reported as mg of gallic acid equivalents per gram of dried product (mg GAE/g dry mass) (Supplementary Fig. 2) showed no significant differences when a fresh and dried sample was compared. Dunnett test (p = 0.717) indicated no change in total phenols content with a value of 28.488 mg GAE/g dry mass for the dried marigold sample (Table 1). There are some reports of the total phenolic content of dried marigolds by different methods [23] passing from 55.8 mg GAE/g (fresh sample) to values between 43.6 and 60.0 mg GAE/g DW where the increase in this parameter was observed when combined far-infrared radiation with hot air convection was applied. Dorozko [34] reported an increment in total phenolic content from 1058 to 1062 mg GAE 100 g^−1^ DW and 1122 mg GAE 100 g^−1^ DW in marigold flowers petals by using freeze drying and microwave drying, respectively. Differences between phenols content in the present study and the reported one are attributed to the stage of maturity, agricultural practices (genetics, fertilizers, insolation, and irrigation), and postharvest handling of the marigold. In general, it was possible to observe that solar drying allowed the preservation of phenols, which are important compounds that provide antioxidant activity and have gained more attention in recent years as potential agents for preventing and treating several oxidative stress-related and chronic diseases [35]. Akshaya has reported the changes in total phenolic content in marigolds [31].

### Total Flavonoid Content

Flavonoids are reported to have a potential role in the prevention of cardiovascular diseases, prevention of age-related neurodegenerative ailments, inactivation of carcinogens, inhibition of cell proliferation, enhancement of DNA repair processes, and reduction of oxidative stress [36], hence the importance in the retention of these compounds after drying (Supplementary Fig. 3). Dunnett test (α = 0.05) showed no significant differences between fresh (6.348 QE/g) and dried (6.622 QE/g) (Table 1) samples, which represents an advantage of solar drying method in the retention of such an important compound of marigold flowers. Gargi [26] reported a decrease in total flavonoid content in fresh pumpkin flower (17.95 µgQE/ml) as compared with tray drying (4.31 µgQE/ml), shade drying (5.22 µgQE/ml), microwave drying (2.95 µgQE/ml), and sun drying (1.59 µgQE/ml). Dorozco [34] reported a decrease in total flavonoid content in marigolds (2522 mg 100 g^−1^ DW) as compared with freeze drying (1947 mg 100 g^−1^ DW), hot air drying (1903 mg 100 g^−1^ DW), and microwave drying (1965 mg 100 g^−1^ DW); according to their report, high temperatures and extended drying times decrease the total flavonoid content.

### Color

**Fig. 3:**
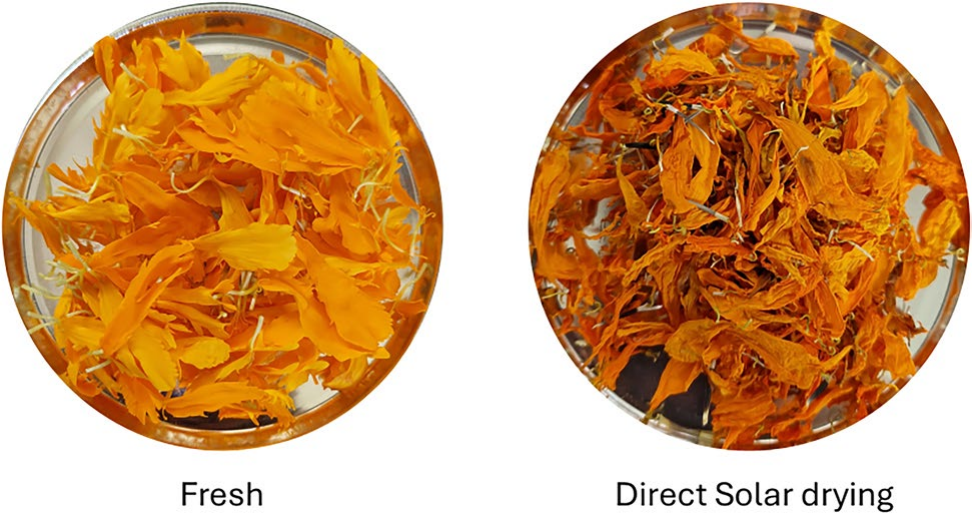
Fresh and dried marigold flower (*Tagetes erecta* L.)

The results of the color analysis indicated a significant difference in luminosity, represented by the *L* parameter. After the solar drying process, a reduction in the luminosity levels was observed with a value of ΔL of −15.51. The color parameter L is reported to be closely correlated with darkening in fruit and vegetable tissues due to enzymatic and nonenzymatic browning after drying [37]. Δa parameter registered a negative value of −1.56. In this case, when Δa adopts negative values, the color tends to be less red. Δb parameter also was negative with a value of −17.5, and this behavior corresponds to a tendency of the dried product to be less yellow. Negative values of Δc (−16.57) for dried petals described that samples resulted in less saturated or with lower intensity compared to the original standard, and negative values of ΔH (−5.85) indicated a shift from yellow to red (Fig. 3), as occurred in the drying of carambola fruit [24], reporting a ΔH from −9.92 to −7.29. ΔE parameter that measures the global color difference took a value of 23.44. In this case, when values exceed a ΔE of 12 describe a pronounced difference according to Hii and Law [38]. Siriamompun [23] reported the changes in the color of marigold flowers by different drying methods; according to their report, the total color difference was 24.2 for freeze drying, 24 for hot air drying, and 19.1 for FIR-hot air drying.

## Conclusions

In the present study, marigold flower petals were dried in a mixed-mode solar dryer due to the highly perishable nature of the flowers and the potential uses that dried products can bring in terms of bioactive compounds and antioxidant properties. After solar drying, it was possible to observe an increment in some properties such as ascorbic acid, from 27.86 to 75.675 mg/100 g; carotenoids, from 2.073 to 18.947 mg/g and antioxidant activity, from 93.51 to 98.18%. This release of bioactive compounds after drying can be advantageous for industrial applications such as food additives, colorants, or pigmenting agents. Also, the main advantage of the present study is the use of sustainable technologies that generate no impact on the environment, and it is easy to use, representing an alternative to conventional drying methods and also an alternative to give a second use to the flowers that usually are treated as a waste after festivities or which is little used the rest of the year.

## Supplementary Information

Below is the link to the electronic supplementary material.

Supplementary file1 (DOCX 278 KB)

## Data Availability

No datasets were generated or analysed during the current study.
