# Supplementary material for: Total Phenolic, Flavonoid Content, and Antioxidant Activity of Dried Marigold (Tagetes erecta L.) Petals Produced in a Mixed-Mode Solar Dryer
Source: Plant Foods Hum Nutr. 2025 May 17;80(3):123. doi: 10.1007/s11130-025-01366-z (PMC12085363; doi:10.1007/s11130-025-01366-z)
Supplement: Supplementary file 1 — Supplementary file1 (DOCX 278 KB) [file 11130_2025_1366_MOESM1_ESM.docx]

**SUPPLEMENTARY INFORMATION**

**Total phenolic, flavonoid content, and antioxidant activity of dried marigold (*Tagetes erecta*** **L) petals produced in a mixed mode solar dryer**

Alfredo Domínguez-Niño ^a,b^, Paulina Guillén-Velázquez ^a,c^, Iris Santos-González^d^, Octavio García-Valladares ^a*^, José Manuel Vázquez-Morales^a^

^a^Instituto de Energías Renovables-UNAM, Departamento de Sistemas Energéticos, Temixco, Morelos, México.  ^b^Secretaría de Ciencia, Humanidades, Tecnología e Innovación, Dirección Adjunta de Desarrollo Científico Mexico City, México. ^c^Secretaría de Ciencia, Humanidades, Tecnología e Innovación, Estancias Posdoctorales por México, Mexico City, México. ^d^Secretaría de Ciencia, Humanidades, Tecnología e Innovación, Investigadoras e Investigadores por México.

*Corresponding author: ogv@ier.unam.mx (O. García-Valladares)

Alfredo Domínguez Niño http://orcid.org/0000-0001-5411-8264

Paulina Guillén Velázquez <https://orcid.org/0000-0002-4508-1102>

Iris Santos González https://orcid.org/0009-0008-1063-5583

Octavio García Valladares <http://orcid.org/0000-0001-9478-4157>

José Manuel Vázquez Morales https://orcid.org/0009-0004-2634-5151

**Materials and methods**

***Fresh marigold samples***

The marigold flower was cleaned, and the pedicel, receptacle, and sepals were removed. Then, the flowers were placed on the dryer trays. The raw marigold flower's initial weight and moisture content were 50 ± 2.0g and 87.35 %, respectively. Samples were placed in a Teflon mesh and introduced into the solar dryer. The marigold flower was obtained in a traditional market of agricultural products in Hueyapan, Morelos, Mexico.

***Dryer description***

For this research, an easy to use and cheap mixed-type was used; it has an integrated photovoltaic fan to favor the humidity drag; the dehydrator can directly absorb the heat of the sun in the drying chamber, and the process is improved by adding an air heater flat plate solar collector. The drying chamber contains ten trays (0.63 x 0.43 m) in five levels; each tray has 0.0025 x 0.007 m perforations. The cover of the drying chamber was made of polycarbonate of 6 mm with ultraviolet protection.

The mixed-type solar dryer has three fundamental parts, a flat plate solar collector for air heating, a drying chamber with a polycarbonate cover and a small photovoltaic fan. The drying chamber can allow the passage of solar radiation through a transparent polycarbonate cover operated in mixed mode or can be covered to operate in indirect mode (only with the hot air coming from the solar collector); inside the drying chamber (0.32m^3^) there are 10 perforated trays of 63 x 43 cm, with a total drying area of 2.7 m^2^, which are arranged in 5 ascending vertical levels. The solar collector for air heating has a gross area of 1.9 m^2^ (1.2 x 1.59 m) with a solar tempered glass of 3.2 mm and an aluminum sheet with selective surface. The air is heated by the solar collector which flows in a combination of natural and forced convection (due to the small photovoltaic fan) into the drying chamber, then the air flows between marigold petals and through processes of heat and mass transfer, the air gives heat to the marigold petals, and they give moisture to the air. Subsequently hot and humid air leaves the drying chamber through the upper part of the drying chamber.

***Drying kinetics***

The solar drying process of marigold flower was carried out on the day: 21^st^ October, 2024 and 24^th^ October, 2024 by using a mixed-type solar dryer (Figure 1).

The moisture content evolution was calculated by applying the equation reported by Vijayan [1]

$M_{wb}=\frac{m_{w}}{m_{w}+m_{d}}=\frac{m_{w}}{m_{t}}$ (1)

Where:

$M_{wb}$ Is the moisture wet basis (kg)

$m_{w}$ Is the mass of water evaporated (kg)

$m_{d}$ Is the dry mass (kg)

$m_{t}$ Is the total mass (kg)

A)


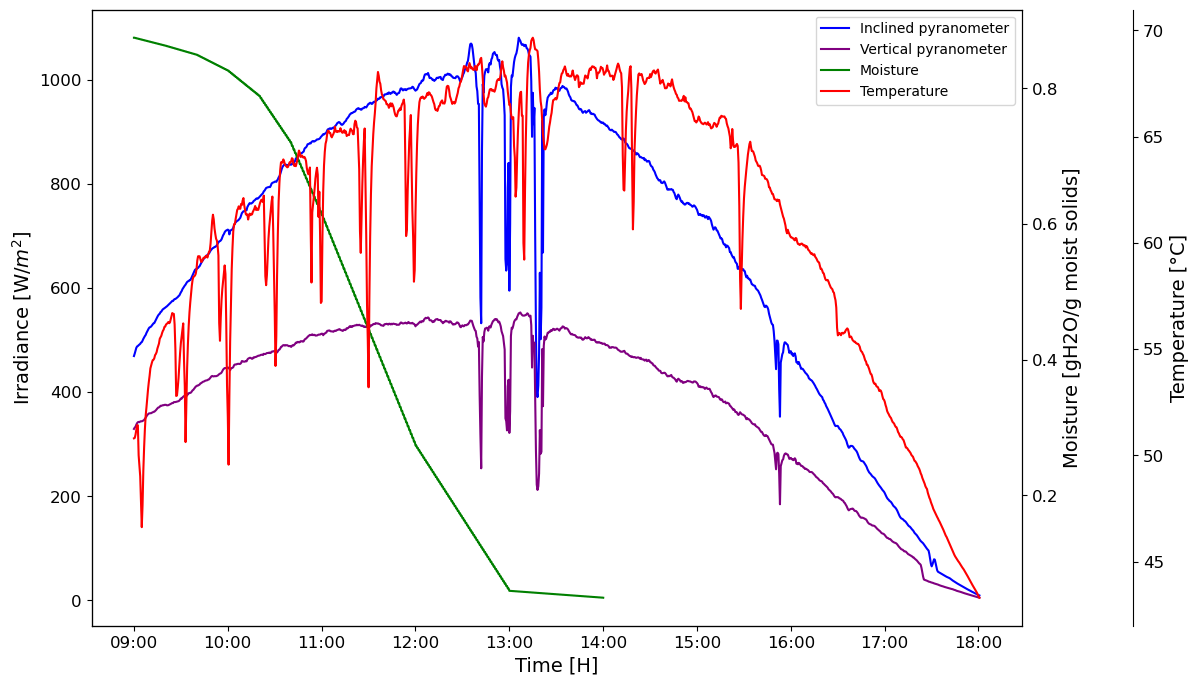


B)


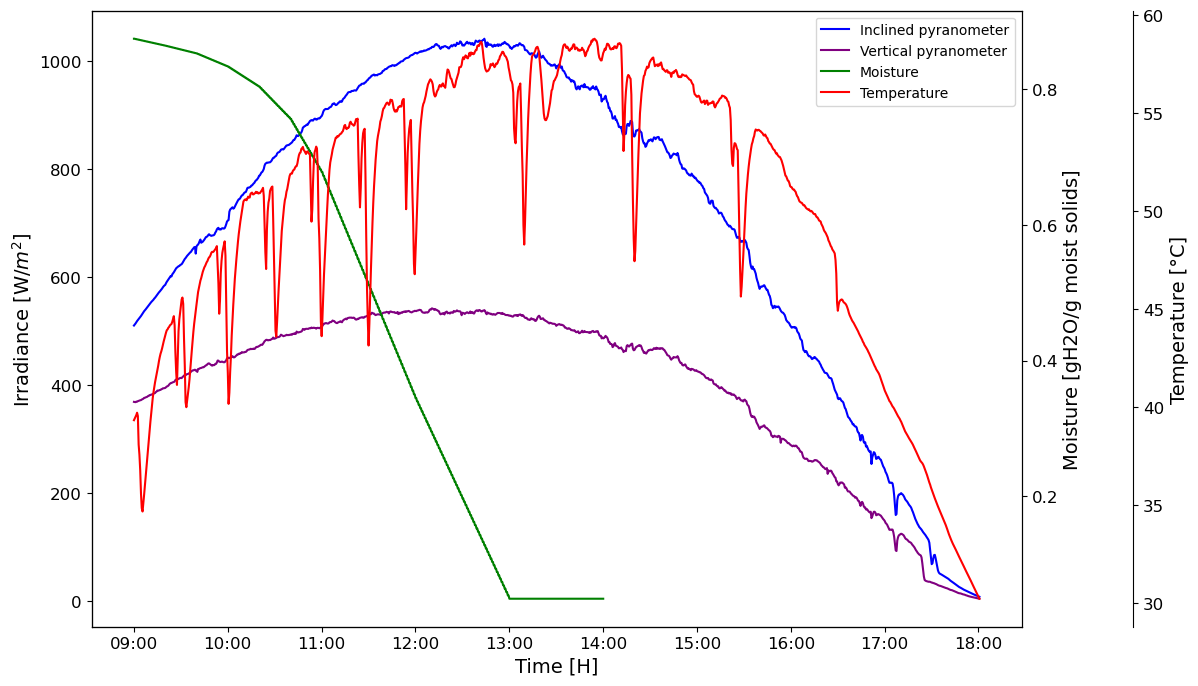


Figure. 1 Drying kinetics of Marigold (*Tagetes erecta* L) carried out in a mixed-type solar dryer, the day: A) 21^st^ October, 2024, B) 24^th^ October, 2024

***Experimental analysis***

The experimental analysis was conducted in triplicate, and the data were analyzed using MINITAB 19. The response variables were moisture content, water activity, color, antioxidant activity, ascorbic acid, total phenols content, total flavonoids content, and carotenoid content, of the dried product.

***Analytical methods***

The moisture content was obtained using a thermobalance (OHAUS, MB45, with a readability of 0.001 g) at 105 °C; around 3 g of the sample was placed and distributed uniformly on an aluminum pan inside the equipment. Water activity (aw) was determined with a Rotronic water activity meter (Higrolab C1) at 25 °C, where the disposable sample cup was covered entirely and introduced inside the kit for 20 minutes. The equipment was calibrated using Rotronic verification standards. Finally, the color properties were determined with a High-Quality Colorimeter (NR60CP+) CIE L a b C H. The values were expressed as L (lightness), a (red-green), b (yellow-blue), H (hue angle), and C (chroma-saturation). From the results obtained, it was possible to calculate the color difference (∆E) between the raw and dried samples, chroma, and hue angle by using the following equations:

| $\Delta E = \left( {\Delta L}^{2}+ {\Delta a}^{2}+ {\Delta b}^{2} \right)^{\frac{1}{2}}$ (2) |  |
| --- | --- |
| $C = \sqrt{{(a)}^{2} + {(b)}^{2}}$ (3) |  |
| $H = arctg\left( \frac{b}{a} \right)$ (4) |  |

The antioxidant content was determined following the methodology proposed by Kailey et al [2]. with slight modifications. The DPPH radical (2,2-diphenyl-1-picrylhydrazyl) inhibition capacity was measured by adding 25 mL of 80% methanol to a 0.5 g sample of the dehydrated herbs. The mixture was left to stand for 40 minutes, after which a 0.1 mL aliquot was taken and combined with 3.9 mL of DPPH radical solution. The test tube was kept in darkness for 30 minutes, and absorbance was measured at 517 nm using a spectrophotometer (Genesys 50 UV Visible Spectrophotometer, Thermo Scientific USA). A blank was prepared using 80% ethanol. The DPPH free radical inhibition (%) was calculated using the following equation:

$\% Inhibition=\frac{Control absorbance- Sample absorbance}{Control absorbance} x 100$ (4)

Ascorbic acid was quantified following the methodology described by García-Valladares et al. [3] by iodometric titration. The Folin-Ciocalteu method was used to determine the total phenols content of marigold flower (Figure 2). It was developed by adding 0.5 mL of the samples and 0.5 mL of Folin-Ciocalteu (2M) reagent. It was well mixed and then left to stand for 10 min after which 1.5 mL of 20% sodium carbonate solution was added. The solution was mixed and incubated at room temperature in the dark. Afterwards, the absorbance was read at 760 nm. The total phenolic contents were expressed in terms of gallic acid equivalent (mg GAE/g of dry mass), a common reference compound. Distilled water was used as blank.

Figure 2. Standard curve of phenol content

Aluminum chloride colorimetric method was used for flavonoids determination (Figure 3). Each plant extract (0.5 ml of 1:10 g/ml) in methanol was separately mixed with 1.5 ml of methanol, 0.1 ml of 10% aluminum chloride, 0.1 ml of 1 M potassium acetate, and 2.8 ml of distilled water. It remained at room temperature for 30 min; the absorbance of the reaction mixture was measured at 415 nm. The calibration curve was prepared by preparing quercetin solutions at concentrations of 20 to 100 mg/mL in methanol (Figure 3).

Figure 3. Standard curve of flavonoid content

Carotenoid content was determined by weighting 0.1 g of sample and dissolving it in 10 mL of acetone in a volumetric flask. The mixture was stirred for 10 min and then vortexed at 5000 rpm during 10 min. The supernatant was collected, and absorbance was measured at 472 and 508 nm using a UV-Vis spectrophotometer (Genesys 50 UV Visible Spectrophotometer, Thermo Scientific USA). Carotenoid essays were determined in triplicate, and calculations were carried out using the equations showed below:

$C^{R}=\frac{A_{508}*2144.0-A_{472}*403.3}{270.9}$ (5) $C^{Y}=\frac{A_{472}*1724.3-A_{508}*2450.1}{270.9}$ (6)

$C^{T}=C^{R}+C^{Y}$ (7)

Where C^R^ represents the red isochromatic fraction content (μg/mL), C^Y^ represents the yellow isochromatic fraction content (μg/mL), and C^T^ is the total carotenoid content (μg/mL).

**References**

1. Vijayan S, Arjunan T, Kumar A (2017) Fundamental concepts of drying. In: Om P, Anil K (eds) Solar drying technology, 1st edn. Springer, Singapore, pp 3-38
2. Kailey R, Dhawan K, Rasane P, Singh J, Kaur S, Singh BP, Kaur D (2019) Utilization of Foeniculum vulgare in herbal candy preparation and analysing its effect on the physico-chemical and sensory properties. Current Science, 116. DOI:10.18520/cs/ v116/i12/2013-2019
3. García VO, Cesar MAL, López VEC, Castillo TB, Ortiz SCA, Lizama TFI, Domínguez NA (2022) Effect by using a modified solar dryer on physicochemical properties of carambola fruit (*Averrhoa Carambola* L.). Rev Mex Ing Quím. 21-Alim2650. https://doi.org/10.24275/rmiq/Alim2650
